# Uptake of HIV self‐testing and linkage to treatment among men who have sex with men (MSM) in Nigeria: A pilot programme using key opinion leaders to reach MSM

**DOI:** 10.1002/jia2.25124

**Published:** 2018-07-22

**Authors:** Waimar Tun, Lung Vu, Osasuyi Dirisu, Adekemi Sekoni, Elizabeth Shoyemi, Jean Njab, Sade Ogunsola, Sylvia Adebajo

**Affiliations:** ^1^ HIV and AIDS Program Population Council Washington DC USA; ^2^ HIV and AIDS Program Population Council Abuja Nigeria; ^3^ College of Medicine University of Lagos Lagos Nigeria; ^4^ HIV and AIDS Program Population Council Lagos Nigeria

**Keywords:** HIV, self‐testing, MSM, feasibility, HIV positivity rate, linkage to treatment, Nigeria

## Abstract

**Introduction:**

HIV self‐testing (HIVST) offers an alternative to facility‐based HIV testing services, particularly for populations such as men who have sex with men (MSM) who may fear accessing testing due to stigma, discrimination and criminalization. Innovative HIV testing approaches are needed to meet the goal of 90% of people living with HIV being diagnosed. This study piloted an intervention to distribute oral HIVST kits to MSM through key opinion leaders (KOLs) in Lagos, Nigeria and assessed the feasibility, acceptability, uptake of HIVST and linkage to HIV treatment.

**Methods:**

A cohort study was conducted (May through September 2017) with 319 participants who were recruited by 12 KOLs through their networks. A baseline survey was conducted at the time of the oral HIVST kit (OraQuick^®^ HIV antibody test) distribution to eligible MSM followed by a 3‐month follow‐up survey to assess usage of and experience with the HIVST kits. Each participant was given two kits.

**Results:**

The median age of the participants was 25 years, 88.7% were literate and 17.9% were first‐time testers. Of the 257 participants (80.7% retention) who completed the three‐month follow‐up interview, 97.7% reported using the HIVST kit and 14 (5.6%) self‐reported an HIV positive result. A quarter (22.7%) tested themselves the same day they received the kit, and 49.4% tested within one week. Almost all participants reported that the HIVST kit instructions were easy or somewhat easy to understand (99.6%) and perform the test (98.0%). The most common reasons they liked the test were ease of use (87.3%), confidentiality/privacy (82.1%), convenience (74.1%) and absence of needle pricks (64.9%). All 14 participants who tested positive had sought confirmatory testing and initiated HIV treatment by the time of the three‐month survey.

**Conclusions:**

HIVST distribution through KOLs was feasible and oral self‐testing was highly acceptable among this urban MSM population. Despite concerns about linkage to treatment when implementing self‐testing, this study showed that linkage to treatment can be achieved with active follow‐up and access to a trusted MSM‐friendly community clinic that offers HIV treatment. HIVST should be considered as an additional option to standard HIV testing models for MSM.

## 1 Introduction

HIV testing is critical in reaching the UNAID's 90‐90‐90 goals of having 90% of people living with HIV diagnosed, 90% of them on antiretroviral therapy (ART) and 90% of them virally suppressed 1. However, for key population groups including men who have sex with men (MSM), uptake of HIV testing services is often low due to multilevel factors such as stigma and discrimination, criminalization, and fear of having trouble with law enforcement authorities 2, 3, 4, 5, 6, 7. In Nigeria, not only is same‐sex sexual activity illegal, but the passage of the Same Sex Marriage (Prohibition) Act of 2013 has ushered in more violence against the LGBT community and has greatly restricted activities of nongovernmental organizations providing services to LGBT people, including any kind of health services. In Nigeria, the estimated HIV prevalence among MSM is 23% to 55% 8, 9, 10. Furthermore, only 65% of MSM have ever had an HIV test, and HIV testing rate during the past 12 months is less than 25% 10, 11. To overcome barriers to HIV testing uptake, the World Health Organization (WHO) developed HIV guidelines recommending HIV self‐testing (HIVST) be offered in addition to existing facility‐testing modalities (2016) 12. HIVST does not require presence of a health provider, thus ensuring privacy and enabling convenience that are particularly important to MSM.

Oral HIVST involves the client obtaining an oral specimen by swiping the gums with the test swab, performing the test and interpreting the test result him/herself in private. It is considered a screening test and does not provide a definitive diagnosis; thus, it requires confirmatory testing. HIVST is considered particularly important for MSM who are reluctant to access static or mobile testing services or MSM concerned about disclosure of sexual orientation when seeking HIV services with health providers 13, 14, 15. Globally, studies have found consistently high levels of HIVST acceptability among the general population and higher‐risk subgroups (including MSM) who may not access other testing services 14, 16, 17, 18, 19, 20, 21, 22, 23, 24, 25, 26, 27, 28, 29, 30, 31. In addition, several studies have shown HIVST increases test coverage for both first‐time and repeat testers 14, 18, 19, 20, 32, 33. Citing this early evidence, the 2016 WHO HIVST guidelines recommend HIVST and specifically call for implementation pilots to understand the effect and operational aspects HIVST distribution in a real‐world setting 12. This is more important as national policy makers are hesitant to endorse HIVST without sufficient evidence that it is feasible, cost‐effective, safe, and leads to acceptable levels of linkage to post‐test services (i.e. confirmatory testing and HIV care and treatment).

We conducted an implementation science research (IS) project, assessing the acceptability, feasibility and operationalization of a distribution model whereby key opinion leaders (KOLs) distribute self‐test kits to MSM in Lagos. Specifically, we examined HIVST uptake (usage), HIV positivity rate, linkage to confirmatory testing and HIV treatment, and perceived benefits and potential harms. This project is the first of its kind being implemented in Nigeria, and is the first implementation science research project to examine HIVST among MSM in sub‐Saharan Africa.

## 2 METHODS

### 2.1 Study design

A cohort study was conducted (May to September 2017) in Lagos. A baseline survey was conducted at the time of the kit distribution to eligible MSM followed by a three‐month follow‐up survey with baseline participants to assess usage of and experience with the HIVST kits and access to confirmatory testing and HIV treatment (among those who tested positive).

### 2.2 Study population

Eligible participants were biological males aged 17 to 59 years, reporting anal intercourse with a man in the past 6 months, self‐reporting HIV negative or unknown status, having no recent HIV test (<3 months), having lived in the city of Lagos for the three months prior to the interview, and planning to stay in Lagos for the next four months.

### 2.3 Description of the distribution model

Twelve KOLs were trained to recruit MSM from their own personal networks. These KOLs were selected from a pool of KOLs who had been trained as HIV counselors for the Population Council's MSM‐friendly community‐based health center (CHC) in Lagos. KOLs were selected by the CHC management staff to work for the CHC services because they were highly respected and endorsed by their peers (i.e. were recommended by other MSM clients of the CHC) and influential (i.e. successful in referring many of their peers to the CHC), showed great enthusiasm and motivation to serve as KOLs, and had real‐time information about their MSM community and hotspots (physical and virtual), which were corroborated by other KOLs and the CHC's programmatic staff. KOLs had to be at least 18 years of age, self‐identify as MSM or gay, have completed secondary education and good interpersonal skills, and be able to mention at least ten hotspots where MSM meet. KOLs were diversified in age and professional and educational status: six were older than 25 years and six were younger; three had some or had completed secondary schooling, six were currently in or had completed tertiary education, and three had completed certificate or trade school; and their current professional status was current students (4), part‐time employed (3), full‐time employed (2) and self‐employed (3). KOLs mobilized potential participants at hotspots considered to be safe, such as football fields, gyms and cafes. Virtual mobilization was done via mobile applications (primarily WhatsApp). We also received assistance from another local NGO working with MSM to help introduce their MSM constituents to the study KOLs. This NGO is located in another part of Lagos and tends to attract older and more educated professional MSM. KOLs informed potential participants about the study and provided them with information or referral cards to either meet at the Population Council's CHC or another convenient and safe space for the interview and distribution. MSM who wanted to be part of the study and receive the self‐test kits were then formally screened for eligibility by a research assistant or the KOL in a private place; if eligible, the candidate gave consent and was interviewed by the research assistant. After the baseline interview, all participants were provided with: (1) two HIVST kits (OraQuick Rapid HIV ½ Antibody, OraSure Technologies), a rapid oral fluid test kit approved by FDA to detect HIV antibodies, (2) a referral voucher to access free HIV testing at the CHC, and (3) HIV prevention an HIVST information, and condoms and lubricant. At the time of the distribution, the research assistant explained how OraQuick works without providing detailed instruction or demonstrating how to perform the test. Participants were sent a video demonstration of OraQuick on their phones. It should be noted that KOLs accompanied the research assistants during all baseline study visits as their introduction of the research assistants to the potential participants was necessary to gain the trust of the participant in the research assistant.

Due to mental health concerns and at the request of the ethical review boards, a phone hotline was set up and managed by a certified HTS counsellor for four months to provide information on HIVST kit use, counselling, and referrals for HIV care and treatment and other support services. The hotline information was included in the package given to participants. In addition, the HTS counsellor followed‐up with participants by calling participants at five, 30 and 80 days after participants received the HIVST kits to provide support for usage and facilitate referrals for HIV treatment for those testing positive. In order to ensure that the follow‐up calls did not bias our research (i.e. the follow‐up call may motivate people to use the test), the counsellor was instructed to simply assess the participant's psychological well‐being and not promote the use of the test kit. In the event that the counsellor deemed the participant needed further counselling and support, the counsellor advised the participant to come into CHC to meet with the counsellor for further counselling.

### 2.4 Baseline survey

The baseline interview covered demographic profile, HIV‐related risk behaviours, HIV testing history, reasons for not having tested, and self‐perceived HIV risk. Each interview lasted 35 to 45 minutes. At baseline, the names (or nicknames) and contact information (mobile number) of the participants were collected to ensure successful follow‐up with participants for the three‐month survey. The baseline data collection and HIVST kit distribution was completed within 32 days.

### 2.5 Three‐month follow‐up interviews

A follow‐up interview was conducted after three months to determine whether participants had used the HIVST kits provided and their experience with it, their self‐reported test result, potential harms, confirmatory testing (if positive), linkage to ART, and willingness to pay. Follow‐up interviews took place at the CHC or at a convenient safe location. All participants provided written informed consent and were reimbursed 1500 Nigerian Nairas (approximately USD 4.15) for each the baseline and follow‐up interviews.

### 2.6 Data analysis

Descriptive statistics of the quantitative survey data were computed using STATA Software (Version 14.1, College Station, Texas). Univariate data analysis was used to describe the characteristics of the study population and their perceptions and behaviours related to self‐testing.

### 2.7 Ethical considerations

Ethical approval for the study was obtained from the Population Council Institutional Review Board and the College of Medicine of the University of Lagos Health Research and Ethical Committee.

## 3 Results

### 3.1 Participant characteristics

A total of 686 MSM were reached by KOLs (i.e. made contact via face‐to‐face, phone, text messaging or social media app whereby self‐testing was introduced) of whom 388 were referred to the study for the initial study screening visit. Of those, 307 (79.1%) were referred through a one‐on‐one meeting with a KOL, 27 (7.0%) through one‐on‐one social media contact (e.g. WhatsApp) with a KOL, 24 (6.2%) were referred by a friend or boyfriend, 30 (7.7%) through other means. Of the 388 referred to the study, 63 were deemed ineligible (multiple reasons possible): 25 had not had sex with a man in the previous six months, six had not lived in Lagos the preceding three months or were planning to move from Lagos in the next three months, 15 had tested for HIV in the last three months, 15 had previously tested HIV positive, and 14 were on ARV drugs, and one had previously received a self‐test kit prior to the study. Five people did not provide consent, and one was dropped due to multiple enrolment yielding a sample of 319 participants. A total of 177 (55.5%) were reached for the 5‐day follow‐up call by the counsellor, 218 (68.3%) for the 30‐day call, and 77 (24.1%) for the 80‐day call. Of the 319 participants,

The characteristics of the baseline participants are reported in Table 1. The median age was 25 years and 86.8% were never married. Almost all participants had completed secondary education or higher; 27.9% had completed tertiary education. About one‐quarter (27.0%) were currently enrolled in school or university, and the majority (88.7%) were literate. Most participants self‐identified as bi‐sexual (67.4%) or homosexual (31.0%). Most of the participants had previously tested for HIV; 17.9% had never previously tested for HIV.

**Table 1:** Characteristics and behaviours of baseline participants (N = 319)

Characteristics	n (%)
Median age (IQR)	25 (21, 32)
Marital status	
Never married/single	277 (86.8)
Single but living with male partner	18 (5.6)
Married to a woman	24 (7.5)
Education	
Some secondary or less	14 (4.4)
Completed secondary	133 (41.7)
Some tertiary	83 (26.0)
Completed tertiary	89 (27.9)
Currently in school/university	
Full‐time	74 (23.2)
Part‐time	12 (3.8)
Not enrolled	233 (73.0)
Literacy	
Illiterate	13 (4.1)
Partially literate	23 (7.2)
Literate	283 (88.7)
Sexual self‐identity	
Homosexual	99 (31.0)
Bi‐sexual	215 (67.4)
Straight/heterosexual	4 (1.3)
Not sure	1 (0.3)
Previously HIV tested and received result	
Never	57 (17.9)
More than 1 year ago	115 (36.1)
Tested in last 7 to 12 months	91 (28.5)
Tested within last 6 months	56 (17.5)
Median number of male sex partners in the last six months (IQR)	3 (2, 6)
Type of partner last male partner	
Regular steady partner	180 (56.3)
Casual partner	121 (37.8)
Paying partner	19 (5.9)
Did not use a condom at last sex with a man	84 (26.3)
Self‐perceived likelihood of being HIV positive	
Very likely	26 (8.2)
Somewhat likely	67 (21.0)
Unlikely	107 (33.5)
Very unlikely	119 (37.3)

Participants reported a median of three male sex partners in the last six months; 56.3% reported their last male partner was a regular partner and 37.8% reported a casual partner. A quarter (26.3%) had not used a condom at last sex with a man. A high proportion (29.2%) felt they were very likely or somewhat likely to be HIV positive.

### 3.2 Lost to follow‐up

A total of 257 participants (80.6%) completed the follow‐up interview. In comparing the characteristics of participants, there were no significant differences between the 62 participants lost to follow‐up and those retained in the study with regard to education, sexual identity, HIV testing history, condom use at last sex with a man, and self‐perceived HIV risk. [Data not shown] However, those lost to follow‐up were slightly younger than those retained in the study (<25 years: 64.5% vs 50.6%; *p* < 0.05) and had a higher score on the Beck Depression Inventory (3.8 vs 2.4; *p* < 0.05) 34. Of the 62 participants who were lost to follow‐up, 43 were not reachable by the counsellor on the 80‐day follow‐up call, 10 were reached but did not show up for the interview, four were wrong number or person denied being the participant, two no longer wanted to participate of could not attend the endline visit, and three had missing data on follow‐up calls. Although the 62 participants did not participate in the endline survey, data from the counsellor follow‐up log from the five‐day or 30‐day calls indicates that 40 of them reported having used the test kit; usage was unknown for the remaining.

### 3.3 Use of HIV self‐testing

Each participant received two HIVST kits at baseline. Table 2 describes the self‐reported HIVST behaviour of participants at the three‐month study visit (N = 257). The majority (97.7%) reported having used the kit on themselves. With the second test kit, 36.2% reported testing themselves again, 33.1% kept it for future use, 21.4% gave it to a friend or a family member, and 8.2% gave it to a sex partner. There was no statistical difference in use of second test kit by HIVST test result, HIV testing history, condom use at last sex, and sexual identification. A quarter (22.7%) tested themselves the same day they received the kit, and 49.4% tested within one week. About a quarter (23.5%) reported that they had someone else present while they tested (55.0% with a friend, 21.7% with a family member, 16.7% with a sex partner and 6.7% with a KOL).

**Table 2:** Self‐reported HIV self‐testing (HIVST) behaviour of participants at the 3‐month follow‐up study visit (N = 257)

Variable	n (%)
What they did with the first HIVST kit	
Tested using HIVST kit	251 (97.7)
Kept it for my future use	4 (1.5)
Gave it to a friend	2 (0.8)
What they did with second HIVST kit	
Tested self again	93 (36.2)
Kept it for future use	85 (33.1)
Gave to friend/family member	55 (21.4)
Gave it to sex partner	21 (8.2)
Other	3 (1.2)
Self‐tested how soon after receiving HIVST kit (n = 251)	
Same day	57 (22.7)
Within 1 week	124 (49.4)
Between 1 to 2 weeks	41 (16.3)
More than 2 weeks	29 (11.6)
Someone else present while testing	59 (23.5)
Person present while testing (n = 60)	
Friend	33 (55.0)
Sex partner/spouse	10 (16.7)
Family member	13 (21.7)
Peer educator	4 (6.7)

### 3.4 Perceptions of HIV self‐testing

Table 3 describes the perceptions of HIVST among participants who used the kit (N = 251). Almost all reported that the instructions were easy (92.4%) or somewhat easy to understand (7.2%) and it was easy (90.0%) or somewhat easy (8.0%) to perform the test. Users were asked to indicate what they liked about the HIVST kit. The most common reasons they liked the test were ease of use (87.3%), confidentiality/privacy (82.1%), convenience (74.1%), and absence of needle pricks (64.9%). The majority of users (88.5%) indicated that there was nothing they did not like about the test. Reasons for not liking the test among users (from open‐ended questions of the questionnaire) included: “*The liquid content might pour if one is not careful, something should be done about it*”; “*Just because anyone that sees it eventually during the testing process will know ones result”*; “*A physical counsellor should be attached during the distribution so that the respondents could have access to him; this will help check against suicide tendency*” and “*On the pack it was directly stated that it's for HIV, other mild terms can be used that won't arouse any discriminatory suspicion*.”

**Table 3:** Perceptions of HIV self‐testing (HIVST) among participants who used the test kit (N = 251)

Variables	n (%)
Ease of understanding HIVST instructions	
Easy	232 (92.4)
Somewhat easy	18 (7.2)
Somewhat difficult	1 (0.4)
Difficult	0
Performing the HIVST was:	
Easy	226 (90.0)
Somewhat easy	20 (8.0)
Somewhat difficult	4 (1.6)
Difficult	1 (0.4)
What participants liked about the HIVST kit	
Easy to use	219 (87.3)
Guarantees confidentiality/privacy	206 (82.1)
Convenient to use	186 (74.1)
No need for needle prick	163 (64.9)
Learn test result quickly	145 (57.8)
Easy to understand instructions	138 (55.0)
Easy to interpret results	140 (55.8)
Do not have to go to facility/Do at home	137 (54.6)
Saves time	126 (50.2)
Can test at home with friend/partner	108 (43.0)
Do not have to talk to health provider	96 (38.3)
What participants disliked about the HIVST kit	
Nothing	222 (88.5)
Satisfaction with HIVST experience	
Very satisfied	229 (91.2)
Somewhat satisfied	18 (7.2)
Not satisfied	4 (1.6)
Would use the HIVST kit again in the future	250 (99.6)
Confidence in using the HIVST correctly in the future	
Very confident	219 (87.2)
Confident	30 (12.0)
Somewhat confident	2 (0.8)
Confidence in reading the HIVST result correctly in the future	
Very confident	213 (84.9)
Confident	36 (14.3)
Somewhat confident	2 (0.8)
Would recommend HIVST kit to others	242 (96.4)

The majority (91.2%) were very satisfied with the self‐testing experience, almost all the participants reported that they would use the kit again in the future (99.6%), and would recommend the HIVST kit to others (96.4%). They also were very confident that they could use the test (87.2%) and read the result (84.9%) correctly in the future.

### 3.5 Linkage to HIV care and treatment

Table 4 describes the result of the self‐tests and linkages to care and treatment among the 251 users of the test. A total of 14 participants (5.6%) self‐reported that they tested positive using the HIVST kit, 7 (2.8%) had indeterminate or invalid results, and four (1.6%) were not sure of their result. Of the seven who reported indeterminate or invalid result, two sought HIV testing and of the four who were unsure of their result, one sought HIV testing afterwards. Over one‐half (60.2%) told someone else the test result (friend 43.1%, sex partner 23.8%, counsellor 20.5%, family member 14.6%, KOL 13.9%, health worker 3.3%).

**Table 4:** Result of HIV self‐testing (HIVST) and linkage to HIV care and treatment among participants who used the HIVST kit (N = 251)

Variable	n (%)
Result of HIVST	
Negative	226 (90.0)
Positive	14 (5.6)
Indeterminate/Invalid	7 (2.8)
Not sure	4 (1.6)
Told someone the test result	151 (60.2)
Who participant told of HIVST result (n = 151)	
Friend	65 (43.1)
Sex partner	36 (23.8)
Counsellor	31 (20.5)
Family member	22 (14.6)
Peer educator	21 (13.9)
Health worker	5 (3.3)
Sought HIV counselling after learning:	
HIV negative result (n = 226)	25 (11.1)
HIV positive result a(n = 14)	14 (100.0)
Indeterminate/Invalid (n = 7)	2 (28.6)
Not sure (n = 4)	1 (25.0)
Thought of harming self due to positive result (n = 14)	1 (7.1)
Confirmed registered for HIV treatment (n = 14) a	14 (100.0)
Confirmed started HIV treatment (n = 14)^a^	14 (100.0)

a Post‐test counselling, and registration and initiation of HIV treatment was based on the counsellor's follow‐up with the HIV positive participants and confirmation against the Community Health Centre's patient records.

Among the 226 participants who tested negative, 11.1% sought HIV counselling and testing after self‐testing. The reasons given for not seeking post‐test counselling were that they were negative and did not see the need for counselling (85.7%) and they knew how to maintain their negative status (38.6%). Among the 14 who tested positive, all sought post‐test counselling and had their test results confirmed at the CHC following the national testing algorithm, that is, all 14 were confirmed HIV positive and all accepted and have initiated HIV treatment. This information was confirmed through the CHC.

Only nine participants called into the helpline; five of the nine callers had tested positive and were seeking support.

### 3.6 Preference for where to obtain HIV self‐testing kits

**Figure 1:**
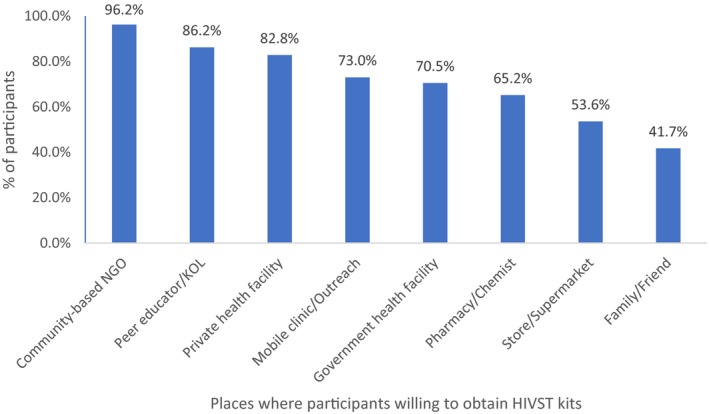
Places where participants were willing to obtain HIV self‐testing (HIVST) kits (N = 319)

Figure 1 describes places where participants at baseline indicated they would be willing to obtain HIVST kits. The most acceptable place was community‐based organization (CBO)/NGO (96.2%) followed closely by peer educators or KOLs (86.2%) and private health facilities (82.8%). When participants were asked to select the one place where they would like to obtain the self‐test kits, CBOs/NGOs (55.9%) was the most commonly selected response followed by private (12.5%) and government (11.6%) health facilities. Reasons proffered for selecting CBOs (from open‐ended questions of the questionnaire) included: “*They understand my sexuality*”, “*No stigma*”; “*This is the only place I can be myself*”; “*I will not be judged for being homosexual*,” and “*They also offer good counselling*.”

### 3.7 Willingness to pay for HIV self‐testing

The majority (85.6%) of participants who received the test kits were willing to pay for the kit. The median maximum participants were willing to pay was N2000 Nigerian Naira (approximately USD 5.50) [Data not shown].

## 4 Discussion

While previous studies have reported on the HIVST acceptability among the general population and MSM 14, 16, 17, 18, 19, 20, 21, 22, 23, 24, 25, 26, 27, 28, 29, 30, 31, this is one of the first studies to report on actual HIVST usage, positivity rate, and linkage to HIV treatment among MSM in Africa. This study showed that distribution of oral HIVST kits to MSM through networks of KOLs is feasible. The high usage rate of the oral HIVST kit clearly highlights the importance and the need for alternative strategies to HIV testing among MSM in Nigeria. It was encouraging that we observed almost universal uptake of the test kits, including 17.9% who were first‐time testers. Lastly, this study showed a high rate of linkage to HIV treatment among this population. The findings from this study are directly responsive to the WHO's 2016 Guidelines on HIVST that calls for furthering the evidence base of HIVST among key populations, especially MSM and groups that have sub‐optimal rate of HIV testing in Sub‐Saharan Africa 12. This initial evidence will help guide the Ministry of Health of Nigeria in introducing and scaling up HIVST as another official testing modality to increase uptake of HIV testing among MSM and potentially other high‐risk and marginalized populations, among whom HIV testing rates are often low.

The distribution of HIVST kits through KOLs was efficient in reaching MSM for self‐testing. A total of 12 KOLs managed to reach a high number of MSM within a fairly short time period (32 days). Feedback from KOLs indicated that it was easy to reach their closest peers, however, it became harder to recruit once they saturated their networks, which is a common occurrence with network sampling 35. KOLs indicated that they were able to recruit additional MSM by relying on their peers to help recruit other peers. The population reached is a fairly high‐risk population (high number of sex partners, type of sex partner, unprotected sex, and high self‐perceived risk of HIV). This study managed to recruit MSM who were first‐time testers (17.9%), which is noteworthy given the intensity of HIV testing programmes targeting MSM by various NGOs in Lagos. This rate is similar to the CHC's community‐based HIV testing activity. While it is indeed important to attract first‐time testers, self‐testing is also an important option for the MSM population, particularly those at continued risk for HIV among whom retesting is critical.

While studies globally, including countries in Africa, have shown high acceptability of HIVST using hypothetical scenarios in different populations 16, 17, 18, 19, 20, 21, 22, 24, 25, 29, 30, 31, 36, 37, this study showed high acceptability with usage with the actual test kit. However, it is important to consider other potential explanations for the high uptake. First, the uptake rate may have been high because those who were interested in using the test likely self‐selected to participate in the study. While intention to use the HIVST kit was not a requirement for participation, it is likely that those who agreed to meet with the KOLs and participate in the study were already intending to use the kit and those who did not want to use the test kit declined to even meet with the KOLs. Despite the potential self‐selection into the study of those who intended to self‐test, this study's high uptake of HIVST as well as the high level of willingness to pay for it provides important insight into the need for this alternative method of testing. In addition, the follow‐up calls made by the counsellor to the participants may have encouraged some to use the test kits. However, this may be minimal as nearly three‐quarter of the participants had used the kit within their first week of receipt. Future HIVST implementation programs should keep this cost in mind during the planning stages.

To date, no other studies have reported HIV positivity rates from HIVST among MSM in Africa. This study found an HIV positivity rate of 5.6%. It should be noted that this does not represent the HIV prevalence in this population as we specifically recruited people who had HIV negative or unknown status. The sample is a fairly young sample, which may explain the relatively low positivity rate. Another reason for the low positivity rate may be that the proportion who had a paying partner in this study was lower than the 24%‐55% reported in other studies with MSM in Lagos 8, 38. This positivity rate, however, is similar to the positivity rate (5%) seen among MSM tested through the community‐based HIV testing programme in Lagos from April to September 2017. Thus, HIVST distributed through KOLs appears to reach a similar population with similar positivity rate as community‐based HIV testing; thus, it offers MSM an alternative option for testing. To improve HIV positivity rate, future studies should examine more targeted HIVST distribution such as distribution within the networks of HIV‐positive MSM.

Invalid test results are generally higher among self‐testers. Recent literature has identified several steps in which errors could happen, causing an invalid test result, including sample collection using the swab, handling of the sample swab, and following the procedures 39, 40. In our observation of 16 participants as part of the formative assessment before the distribution of the kits, common errors include not collecting sample correctly, not putting the swab into the bottle correctly, and not waiting enough time (20 minutes) before reading the result [data not shown]. In this current study, we found a fairly high rate of indeterminate or invalid results or uncertainty of result (4.4%), which is higher than what has been reported when subjects performed supervised oral HST after a demonstration of the test kit usage in a rural community in rural Mozambique (5/496 = 1.7%) 41. However, one of the first HIVST validation studies conducted in Kenya in 2013 reported a higher rate of invalid HIV results (37/239 = 15.5%) 40. These findings suggest that directly assisted HST may reduce invalid test result, and the need to have a 24‐hour hotline, or mobile app to better assist testers. It is also important to highlight the need for HIVST testers to seek post‐test counselling when they obtain an indeterminate or invalid result.

In addition to distributing HIVST directly through KOLs, community‐based MSM‐friendly HIV prevention and treatment facilities such as the CHC may be a potential distribution outlet. NGOs were highly preferred as a place for accessing HIVST kits by study participants due to the safe non‐judgmental environment with good quality services. In this study, we gave participants the option to pick up the HIVST kits at the CHC or at an alternate safe and convenient location. This study showed that referral for pick‐up of HIVST kits at a facility is an acceptable option with equally high usage as the directly distributed approach; a high percentage (42.2%) chose to come to the CHC for the HIVST kits. A randomized trial in Zambia (the ZEST study) among female sex workers (FSW) which compared a peer educator direct distribution to coupon referral for pick up at a health facility found that FSWs randomized to the referral arm had similar HIVST usage and linkage to care and ART initiation rates as the direct distribution arm (assessed after four months) 42. Thus, future research and implementation should include health facilities as a distribution site as it may be a less costly model of distribution than using KOLs/peer educators. In addition, future research should be conducted to understand potential differences in risk profiles, HIVST uptake, HIV positivity rate, and linkage to HIV care and treatment among those receiving HIVST through different distribution.

Although the helpline was available, only a few participants called into the helpline. This is not surprising as most people found the test instructions easy to understand and the test easy to perform. Other studies have also reported low usage of the helpline 43, 44. Despite low usage, this feature may be worth retaining as it appeared to be most useful for study participants who tested positive in order to obtain support – over half the callers were those who tested positive, and it presents an important opportunity for referral to HIV treatment.

A key concern with self‐testing is whether self‐testers would seek HIV treatment 45, 46. This study demonstrated a noteworthy 100% linkage to HIV treatment; the national linkage rate is 31% 47. The high linkage to treatment in this study is likely due to follow‐up calls by the counsellor after HIVST distribution, the participant's access to their KOLs, and most importantly, the linkage to a well‐trusted MSM‐friendly facility that offers not only HIV prevention services but also HIV treatment. Studies in low‐resource settings among MSM have reported both fairly low 48, 49 and high 50, 51 linkage to HIV care and treatment after self‐testing.

The study has several limitations. First, key outcomes including HIVST uptake and test result were self‐reported. Participants may have over‐reported HIVST uptake and may also be reluctant in reporting a positive test result and thus overestimate the uptake and underestimate HIV positivity rates. However, it is important to note that for those who reported a positive result, we were able to confirm their HIV positive status with confirmatory testing and their linkage to ART by the CHC programme staff. Second, we lost about 20% of the original cohort and key outcomes may be different between those retained in and those lost from the cohort. However, uptake of HIVST among those lost to follow‐up was also fairly high – a minimum of 64.5% used it. A comparison of those lost to follow‐up and those retained in the cohort revealed that while the two groups were comparable regarding demographic and risk characteristics, those lost to follow‐up were younger and scored worse on the depression scale. This should be kept in mind for future implementation as those lost to follow‐up may have been more likely to have experienced harm or psychological distress. Furthermore, regardless of our effort to diversify the profiles of the 12 KOLs, our sample was quite young and educated; therefore, findings may not be generalizable to other segments of the MSM population. It is likely that a young population would underestimate the positive rate among MSM. In addition, the findings may not be generalizable to other settings; for example, the HIVST uptake, ease of use, and linkage to care may be lower in a rural setting, among participants with lower education level or those who do not identify as homosexual or bisexual. Lastly, given that the test kits were provided for free and that there was financial compensation for study participation, it is possible that participants may have participated more than once. However, this is highly unlikely as the small team of interviewers and KOLs would have recognized repeat participants during the short data collection period.

## 5 Conclusion

This pilot study has shown that HIVST is highly acceptable to urban MSM, the majority of whom are mostly educated and self‐identified homosexual or bi‐sexual, and distribution through KOLs is feasible. Furthermore, linkage to HIV treatment can be achieved given active follow‐up and access to MSM‐friendly HIV treatment facilities. While further research is needed to compare outcomes of testing uptake, frequency of re‐testing, HIV‐positive rate, and linkage to care between different HIVST distribution models as well as in comparison to standard HIV testing strategies, this study supports the feasibility and acceptability of HIVST among MSM. HIVST should be considered as an additional option to standard HIV testing models.

## Funding

The research reported in this publication was supported by the U.S. National Institutes of Health under award number 1R21AI124409‐01.

## Disclaimer

The content is solely the responsibility of the authors and does not necessarily represent the official views of the U.S. National Institutes of Health.

## Authors’ contributions

WT, SA and LV conceptualized the study design. LV assisted with protocol and tool development, quantitative data analysis and contributed to manuscript writing. JN assisted with implementation oversight and manuscript review. ES managed day‐to‐day implementation of the intervention and data collection, and contributed to the manuscript writing and review. OD trained study staff and KOLs, supervised data collection and contributed to the manuscript writing and review. AS contributed to the protocol development, training of study staff, manuscript writing. WT trained study staff and KOLs, conducted the data analysis and led the manuscript writing.

## Competing interests

All authors report no competing interests.

## References

[1] UNAIDS . 90‐90‐90: an ambitious treatment target to help end the AIDS epidemic . Geneva, Switzerland:UNAIDS; 2014.

[2] Schwartz SR , Nowak RG , Orazulike I , Keshinro B , Ake J , Kennedy S , et al. The immediate effect of the Same‐Sex Marriage Prohibition Act on stigma, discrimination, and engagement on HIV prevention and treatment services in men who have sex with men in Nigeria: analysis of prospective data from the TRUST cohort. Lancet HIV. 2015;2(7):e299–306. 26125047 10.1016/S2352-3018(15)00078-8 PMC4481876

[3] Vu L , Tun W , Sheehy M , Nel D . Levels and correlates of internalized homophobia among men who have sex with men in pretoria. South Africa. AIDS Behav. 2012;16(3):717–23. 21484279 10.1007/s10461-011-9948-4

[4] Fay H , Baral SD , Trapence G , Motimedi F , Umar E , Iipinge S , et al. Stigma, health care access, and HIV knowledge among men who have sex with men in Malawi, Namibia, and Botswana. AIDS Behav. 2011;15(6):1088–97. 21153432 10.1007/s10461-010-9861-2

[5] Wei C , Cheung DH , Yan H , Li J , Shi LE , Raymond HF . The impact of homophobia and HIV Stigma on HIV testing uptake among Chinese men who have sex with men: a mediation analysis. J Acquir Immune Defic Syndr. 2016;71(1):87–93. 26334742 10.1097/QAI.0000000000000815 PMC4713338

[6] Cloete A , Kalichman SC , Simbayi LC . Layered Stigma and HIV/AIDS: experiences of men who have sex with men (MSM) in South Africa In Liamputtong P, editor. Stigma, Discrimination and Living with HIV/AIDS. Dordrecht: Springer; 2013 p. 259–69

[7] Risher K , Adams D , Sithole B , Ketende S , Kennedy C , Mnisi Z , et al. Sexual stigma and discrimination as barriers to seeking appropriate healthcare among men who have sex with men in Swaziland. J Int AIDS Soc. 2013;16(3 Suppl 2):18715. 24242263 10.7448/IAS.16.3.18715 PMC3833105

[8] Vu L , Adebajo S , Tun W , Sheehy M , Karlyn A , Njab J , et al. High HIV prevalence among men who have sex with men in Nigeria: implications for combination prevention. J Acquir Immune Defic Syndr. 2013;63(2):221–7. 23406978 10.1097/QAI.0b013e31828a3e60

[9] Keshinro B , Crowell TA , Nowak RG , Adebajo S , Peel S , Gaydos CA , et al. High prevalence of HIV, chlamydia and gonorrhoea among men who have sex with men and transgender women attending trusted community centres in Abuja and Lagos, Nigeria. J Int AIDS Soc. 2016;19(1):21270. 27931519 10.7448/IAS.19.1.21270 PMC5146323

[10] National HIV/AIDS & STI Control Porgramme, Federal Ministry of Health, Nigeria . Integrated Biological and Behavioural Surveillance Survey (IBBSS) 2014. 2014.

[11] Federal Ministry of Health, Nigeria . Integrated Biological and Behavioural Surveillance Survey 2010.

[12] World Health Organization . Guidelines on HIV Self‐Testing and Partner Notification (Supplement to Consolidated Guideline on HIV Testing Services). Geneva, Switzerland: World Health Organization; 2016. 27977094

[13] Vu L , Andrinopoulos K , Tun W , Adebajo S . High levels of unprotected anal intercourse and never testing for HIV among men who have sex with men in Nigeria: evidence from a cross‐sectional survey for the need for innovative approaches to HIV prevention. Sex Transm Infect. 2013;89(8):659–65. 23851190 10.1136/sextrans-2013-051065

[14] Johnson CC , Corbett EL . HIV self‐testing to scale up couples and partner testing. Lancet HIV. 2016;3(6):e243–4. 27240785 10.1016/S2352-3018(16)00044-8

[15] Yan H , Yang H , Raymond HF , Li J , Shi LE , Huan X , et al. Experiences and correlates of HIV self‐testing among men who have sex with men in Jiangsu province. China. AIDS Behav. 2015;19(3):485–91. 25480598 10.1007/s10461-014-0968-8 PMC4359059

[16] Han L , Bien CH , Wei C , Muessig KE , Yang M , Liu F , et al. HIV self‐testing among online MSM in China: implications for expanding HIV testing among key populations. J Acquir Immune Defic Syndr. 2014;67(2):216–21. 24991972 10.1097/QAI.0000000000000278 PMC4162828

[17] Heard AC , Brown AN . Public readiness for HIV self‐testing in Kenya. AIDS Care. 2016:1–5. 10.1080/09540121.2016.1191602 PMC5062035 27256543

[18] Johnson C , Baggaley R , Forsythe S , Van Rooyen H , Ford N , Mavedzenge SN , et al. Realizing the potential for HIV self‐testing. AIDS Behav. 2014;18(Suppl 4):S391–5. 24986599 10.1007/s10461-014-0832-x

[19] Kelvin EA , Cheruvillil S , Christian S , Mantell JE , Milford C , Rambally‐Greener L , et al. Afr J AIDS Res. 2016;15(2):99–108. 27399040 10.2989/16085906.2016.1189442 PMC5453183

[20] Krause J , Subklew‐Sehume F , Kenyon C , Colebunders R . Acceptability of HIV self‐testing: a systematic literature review. BMC Public Health. 2013;13:735. 23924387 10.1186/1471-2458-13-735 PMC3750621

[21] Lippman SA , Périssé AR , Veloso VG , Sullivan PS , Buchbinder S , Sineath RC , et al. Acceptability of self‐conducted home‐based HIV testing among men who have sex with men in Brazil: data from an on‐line survey. Cad Saude Publica. 2014;30(4):724–34. 24896048 10.1590/0102-311x00008913 PMC4138047

[22] Pérez GM , Cox V , Ellman T , Moore A , Patten G , Shroufi A , et al., . I know that i do have hiv but nobody saw me’: oral HIV self‐testing in an informal settlement in South Africa. PLoS ONE. 2016;11(4):e0152653. 27044006 10.1371/journal.pone.0152653 PMC4820175

[23] Sun CJ , Stowers J , Miller C , Bachmann LH , Rhodes SD , Rhodes SD . . Acceptability and feasibility of using established geosocial and sexual networking mobile applications to promote HIV and STD testing among men who have sex with men. AIDS Behav. 2015;19(3):543–52. 25381563 10.1007/s10461-014-0942-5 PMC4359067

[24] Tao J , Li MY , Qian HZ , Wang LJ , Zhang Z , Ding HF , et al. Home‐based HIV testing for men who have sex with men in China: a novel community‐based partnership to complement government programs. PLoS ONE. 2014;9(7):e102812. 25051160 10.1371/journal.pone.0102812 PMC4106852

[25] Tucker JD , Wei C , Pendse R , Lo YR . HIV self‐testing among key populations: an implementation science approach to evaluating self‐testing. J Virus Erad. 2015;1(1):38–42. 26005717 10.1016/S2055-6640(20)31145-6 PMC4439005

[26] Martínez Pérez G , Steele SJ , Govender I , Arellano G , Mkwamba A , Hadebe M , et al. Supervised oral HIV self‐testing is accurate in rural KwaZulu‐Natal, South Africa. Trop Med Int Health, 2016 21(6): 759–67. 27098272 10.1111/tmi.12703

[27] Pai NP , Balram B , Shivkumar S , Martinez‐Cajas JL , Claessens C , Lambert G , et al. Head‐to‐head comparison of accuracy of a rapid point‐of‐care HIV test with oral versus whole‐blood specimens: a systematic review and meta‐analysis. Lancet Infect Dis. 2012;12(5):373–80. 22277215 10.1016/S1473-3099(11)70368-1

[28] Pai NP , Joshi R , Dogra S , Taksande B , Kalantri SP , Pai M , et al. Evaluation of diagnostic accuracy, feasibility and client preference for rapid oral fluid‐based diagnosis of HIV infection in rural India. PLoS ONE. 2007;2(4):e367. 17426815 10.1371/journal.pone.0000367 PMC1838923

[29] Pai NP , Sharma J , Shivkumar S , Pillay S , Vadnais C , Joseph L , et al. Supervised and unsupervised self‐testing for HIV in high‐ and low‐risk populations: a systematic review. PLoS Med. 2013;10(4):e1001414. 23565066 10.1371/journal.pmed.1001414 PMC3614510

[30] Choko AT , Desmond N , Webb EL , Chavula K , Napierala‐Mavedzenge S , Gaydos CA , et al. The uptake and accuracy of oral kits for HIV self‐testing in high HIV prevalence setting: a cross‐sectional feasibility study in Blantyre, Malawi. PLoS Med. 2011;8(10):e1001102. 21990966 10.1371/journal.pmed.1001102 PMC3186813

[31] Green K. , Bao VN , Huong PTT , Son VH , Giang HTT , Ha TT , et al., Is HIV self‐testing acceptable to key populations in Vietnam? Results from a cross‐sectional study of men who have sex with men, female sex workers and people who inject drugs., In 21th International AIDS Conference (AIDS 2016) 2016: Durban, South Africa.

[32] Katz DA , Cassels SL , Stekler JD . Replacing clinic‐based tests with home‐use tests may increase HIV prevalence among Seattle men who have sex with men: evidence from a mathematical model. Sex Transm Dis. 2014;41(1):2–9. 24335742 10.1097/OLQ.0000000000000046 PMC3955208

[33] Herbst JH , Sherba RT , Crepaz N , DeLuca JB , Zohrabyan L , Stall RD , et al. A meta‐analytic review of HIV behavioral interventions for reducing sexual risk behavior of men who have sex with men. J Acquir Immune Defic Syndr. 2005;39(2):228–41. 15905741

[34] Beck AT , Beck RW . Screening depressed patients in family practice: a rapid technic. Postgrad Med. 1972;52:81–5. 4635613 10.1080/00325481.1972.11713319

[35] Gile KJ . Improved inference for respondent‐driven sampling data with application to HIV prevalence estimation. J Am Stat Assn. 2011;106(493):135–46.

[36] Pant Pai N , Behlim T , Abrahams L , Vadnais C , Shivkumar S , Pillay S , et al. Will an unsupervised self‐testing strategy for HIV work in health care workers of South Africa? A cross sectional pilot feasibility study. PLoS ONE, 2013 8(11): e79772. 24312185 10.1371/journal.pone.0079772 PMC3842310

[37] Figueroa C , Johnson C , Verster A , Baggaley R . Attitudes and acceptability on HIV self‐testing among key populations: a literature review. AIDS Behav. 2015;19:1949–65. 26054390 10.1007/s10461-015-1097-8 PMC4598350

[38] Merrigan M , Azeez A , Afolabi B , Chabikuli ON , Onyekwena O , Eluwa G , et al. HIV prevalence and risk behaviours among men having sex with men in Nigeria. Sex Transm Infect. 2011;87(1):65–70. 20820061 10.1136/sti.2008.034991

[39] Peck RB , Lim JM , van Rooyen H , Mukoma W , Chepuka L , Bansil P , et al. What should the ideal HIV self‐test look like? A usability study of test prototypes in unsupervised HIV self‐testing in Kenya, Malawi, and South Africa. AIDS Behav. 2014;18(Suppl 4):S422–32. 24947852 10.1007/s10461-014-0818-8

[40] Kurth AE , Cleland CM , Chhun N , Sidle JE , Were E , Naanyu V , et al. Accuracy and acceptability of oral fluid HIV self‐testing in a general adult population in Kenya. AIDS Behav. 2016;20(4):870–9. 26438487 10.1007/s10461-015-1213-9 PMC4799243

[41] Hector J , Davies MA , Dekker‐Boersema J , Aly MM , Abdalad CCA , Langa EBR , et al. Acceptability and performance of a directly assisted oral HIV self‐testing intervention in adolescents in rural Mozambique. PLoS ONE. 2018;13(4):e0195391. 29621308 10.1371/journal.pone.0195391 PMC5886533

[42] Chanda MM , Ortblad KF , Mwale M , Chongo S , Kanchele C , Kamungoma N , et al. HIV self‐testing among female sex workers in Zambia: a cluster randomized controlled trial. PLoS Med. 2017;14(11):e1002442. 29161260 10.1371/journal.pmed.1002442 PMC5697803

[43] Kalibala S , Tun W , Cherutich P , Nganga A , Oweya E , Oluoch P . Factors associated with acceptability of HIV self‐testing among health care workers in Kenya. AIDS Behav. 2014;18(Suppl 4):S405–14. 24974123 10.1007/s10461-014-0830-z PMC4933836

[44] Ngure K , Heffron R , Mugo N , Thomson KA , Irungu E , Njuguna N , et al. Feasibility and acceptability of HIV self‐testing among pre‐exposure prophylaxis users in Kenya. J Int AIDS Soc. 2017;20(1):21234. 28362073 10.7448/IAS.20.1.21234 PMC5467615

[45] Bain LE , Ditah CM , Awah PK , Ekukwe NC . Ethical implications of HIV self‐testing: the game is far from being over. Pan Afr Med J. 2016;25:114. 28292077 10.11604/pamj.2016.25.114.8303 PMC5325486

[46] Johnson CC , Kennedy C , Fonner V , Siegfried N , Figueroa C , Dalal S , et al. Examining the effects of HIV self‐testing compared to standard HIV testing services: a systematic review and meta‐analysis. J Int AIDS Soc. 2017;20(1):21594. 28530049 10.7448/IAS.20.1.21594 PMC5515051

[47] UNAIDS . UNAIDS Data 2017. Geneva, Switzerland 2017.

[48] Ren XL , Wu ZY , Mi GD , McGoogan JM , Rou KM , Zhao Y , et al. HIV care‐seeking behaviour after HIV self‐testing among men who have sex with men in Beijing, China: a cross‐sectional study. Infect Dis Poverty. 2017;6(1):112. 28655340 10.1186/s40249-017-0326-y PMC5488343

[49] Qin Y , Tang W , Nowacki A , Mollan K , Reifeis SA , Hudgens MG , et al. Benefits and potential harms of human immunodeficiency virus self‐testing among men who have sex with men in China: an implementation perspective. Sex Transm Dis. 2017;44(4):233–8. 28282650 10.1097/OLQ.0000000000000581 PMC5347468

[50] Zhong F , Tang W , Cheng W , Lin P , Wu Q , Cai Y , et al. Acceptability and feasibility of a social entrepreneurship testing model to promote HIV self‐testing and linkage to care among men who have sex with men. HIV Med. 2017;18(5):376–82. 27601301 10.1111/hiv.12437 PMC5340630

[51] Volk JE , Lippman SA , Grinsztejn B , Lama JR , Fernandes NM , Gonzales P , et al. Acceptability and feasibility of HIV self‐testing among men who have sex with men in Peru and Brazil. Int J STD AIDS. 2016;27(7):531–6. 25971262 10.1177/0956462415586676 PMC4643427

